# Systemic Treatment and Outcome in Erythrodermic Psoriasis: A Retrospective Multicenter Study

**DOI:** 10.1111/ijd.70422

**Published:** 2026-04-17

**Authors:** Julian Kögel, Sandra Schuh, Julia Welzel, Michael Sticherling, Lukas Sollfrank, Carola Berking, Teodora Pumnea, Valerie Glatzel, Lars French, Felix Lauffer, Martin Valentin Vitus, Tilo Biedermann, Susanne Bauer, Michael Spindler, Mark Berneburg, Dennis Niebel

**Affiliations:** ^1^ Department of Dermatology University Hospital Regensburg Regensburg Germany; ^2^ Department of Dermatology and Allergology University Hospital Augsburg Augsburg Germany; ^3^ Department of Dermatology University Hospital Erlangen Erlangen Germany; ^4^ Department of Dermatology and Allergology Ludwig‐Maximilian‐University Munich Germany; ^5^ Dr. Phillip Frost, Department of Dermatology and Cutaneous Surgery University of Miami, Miller School of Medicine Miami Florida USA; ^6^ Department of Dermatology and Allergology Technical University Munich Munich Germany

**Keywords:** antirheumatic drugs, biologics, comorbidity, drugs, erythroderma, fumarates, Germany, outcome, psoriasis, treatment

## Abstract

**Background:**

Erythrodermic psoriasis (EP) is a rare but severe condition. Because of its low prevalence, there are no standardized treatment recommendations for EP. Specific EP guidelines are outdated, prioritizing conventional disease‐modifying antirheumatic drugs (cDMARDs) and tumor necrosis factor‐alpha (TNF‐α) inhibitors.

**Methods:**

We conducted a multicenter retrospective chart analysis in five academic centers in Bavaria, Germany (Augsburg, Erlangen, LMU Munich, TU Munich, Regensburg). Patients diagnosed with EP between 2019 and 2024 who received systemic treatment were included in the study.

**Results:**

A total of 29 patients were included. cDMARDs were initiated in 8 patients (27.6%). Biologics were used in 21 patients (72.4%). Psoriasis Area and Severity Index (PASI) decreased from 31.9 to 10.8 across all therapies (*p* < 0.001). PASI 75 was achieved with methotrexate (1), cyclosporine (1), fumarates (1), infliximab (1), ustekinumab (2), ixekizumab (1), secukinumab (2), risankizumab (4), and guselkumab (1). PASI 100 was achieved with infliximab (1), ustekinumab (1), and risankizumab (2). Adverse events occurred most frequently in the cDMARDs group.

**Conclusion:**

There is a wide variety of treatment approaches. Standardized guidelines are needed. Biologic therapies, especially interleukin (IL)17 and IL23‐inhibitors, showed favorable outcomes in this cohort and warrant prospective evaluation.

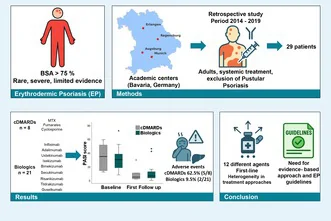

## Plain Language Summary

- Erythrodermic psoriasis (EP) is a rare disease in which at least 75% of the body surface is affected by a chronic disease called psoriasis. Patients experience extensive redness, scaling, and general inflammation. EP may be accompanied by fever and a reduced general condition; therefore, it may become life‐threatening. Unlike psoriasis vulgaris (PsO), research into the pathophysiology of this disease and its treatment options is limited, and the existing guidelines for managing this severe condition are outdated.
- We therefore conducted a retrospective study in five centers in Bavaria, Germany, to collect and analyze data from patients with EP who were treated with systemic therapies commonly used in PsO. The study period was from 2019 to 2024. A total of 29 patients were included. They received 12 different systemic treatments. Therapy had to be discontinued in eight patients. Modern biologic agents showed marked clinical improvement in this cohort, whereas conventional treatments were also effective but more frequently associated with adverse events.
- These findings demonstrate the wide range of treatment approaches, even within a specific geographic location in Germany. Prospective studies and updated treatment guidelines are urgently needed.

## Graphical abstract

This multicentre real‐world study illustrates the heterogeneity of systemic treatment approaches in erythrodermic psoriasis (EP) in Bavaria, Germany. Adverse events were more frequently observed with conventional therapies, whereas biologic therapies showed favorable outcomes. These findings highlight the need for standardized treatment guidelines and further prospective studies.

**Abbreviations**
BSA: Body surface area
cDMARDs: Conventional disease‐modifying antirheumatic drugs
EP: Erythrodermic psoriasis
GPP: Generalized pustular psoriasis
NPF: National Psoriasis Foundation
PsO: Psoriasis vulgaris

## 1 Introduction

Plaque psoriasis (PsO) is a common chronic inflammatory skin disease, affecting about 2%–4% of the general population, with the highest prevalence in northern high‐income countries [1]. Erythrodermic psoriasis (EP) is rare; about 1%–2% of psoriasis patients will eventually develop this manifestation. Contrarily, EP is responsible for up to 25%–40% of cases of erythroderma [2, 3]. The mean age of onset in EP spans from 48 to 55 years, which is notably higher than most cases of PsO, and males are affected more often, with an ethnic propensity for Asians [3, 4].

The pathophysiology of EP is subject to controversy. There is evidence for a T helper (Th)1/Th2 imbalance in EP patients, with levels of immunoglobulin E (IgE) and Th2 cytokines like interleukin (IL)4, IL13, and IL10 higher in EP patients [5, 6, 7]. Yet it is unclear whether the Th2 activity leads to an onset of EP or resolution of the disease [8].

Erythroderma is typically defined as an erythematous involvement of more than 90% of the body surface area (BSA) [9, 10, 11]. In contrast, EP lacks a uniform definition. Although some authors consider EP to be present when more than 75% of BSA is affected, others require an involvement of at least 90% [2, 3, 8]. In this study, EP was defined as involvement of > 75% of BSA in order to include patients with clinically generalized inflammatory disease corresponding to EP, for whom retrospective documentation of BSA may not be accurate.

Morbidity and mortality are increased in EP patients. In a case series of 50 patients with EP, four patients (9%) died, yet only one death could be associated directly with the disease [3]. Green et al. described a series of five patients with EP suffering from staphylococcal septicaemia [12]. In another study, heart failure has been reported in up to 11.1% of EP patients [13].

EP requires targeted and effective treatment. Yet, EP treatment is based on treatment regimens used for PsO. National Psoriasis Foundation (NPF) guidelines are outdated (2010) and propose infliximab, cyclosporine, acitretin, or methotrexate as first‐line therapeutics, with etanercept being mentioned as a second‐line option [14]. Monoclonal antibodies targeting IL17 and IL23, which are considered as first‐line options on the basis of expert opinion, are not included in the NPF guidelines [15, 16]. Because of the lack of evidence, national guidelines for PsO lack specific recommendations for the treatment of EP [17, 18, 19, 20]. Current evidence on EP treatment remains limited and heterogeneous, being largely on the basis of small case series and case reports, whereas real‐world evidence or randomized controlled trials are still missing.

## 2 Materials and Methods

We conducted a retrospective, multicentric study in Bavarian academic centers (Augsburg, Erlangen, LMU Munich, Regensburg, TU Munich). Approval for the research project was granted by the Ethics Committee of the University of Regensburg (project number 24–3742‐104). The positive vote was communicated to the Ethics Committees of the other participating academic centers. Patients with EP who were treated systemically in the participating centers between 2019 and 2024 were included in the study. EP was defined as affected BSA > 75%. Patients of at least 18 years of age were included. A board‐certified dermatologist made the diagnosis of EP, primarily on the basis of clinical and histological findings (where available). Diagnoses were re‐evaluated during the retrospective chart review, and cases in which an alternative diagnosis emerged during follow‐up were excluded accordingly. Cases in which a drug reaction was considered a plausible primary cause of erythroderma were excluded from the analysis. Patients with generalized pustular psoriasis (GPP) or other types of erythroderma were excluded. Clinical and demographic parameters (age group, sex, medication) and psoriasis‐related items (comorbidity, initial diagnosis, histological findings, psoriasis arthritis, previous therapy with conventional disease‐modifying antirheumatic drugs [cDMARDs], biologics, and phototherapy) were evaluated. Medications associated with provoking EP were evaluated. The date of diagnosis of EP with the corresponding systemic therapeutic agent and dosage were recorded. Systemic therapies were prescribed according to the treating physician's discretion at the participating centers, as this was a retrospective observational study without a predefined treatment protocol. Objective follow‐up parameters and objective outcome measures (BSA, Psoriasis Area and Severity Index [PASI], Dermatology Life Quality Index [DLQI]) were recorded, if available. The primary outcome was defined as the change in PASI from baseline to the first available follow‐up. Additionally, outcomes for the subgroup with follow‐up within 8–16 weeks, as well as those achieving PASI75 or PASI100 at any time during follow‐up, are reported separately. PASI outcomes were attributed to the initial systemic treatment started for EP at baseline. Therapy‐associated side effects and possible recurrence of EP during systemic treatment were recorded. Supportive measures such as topical therapy, fluid management, and infection control were part of routine clinical care but were not consistently captured in the retrospective chart analysis. Subsequent treatment discontinuations or switches during follow‐up were documented descriptively. Data were collected individually in the participating centers and analyzed at the University Hospital Regensburg. A descriptive statistical analysis of the entire data set was carried out using IBM SPSS Statistics Version 28 (Armonk, NY, USA). A *p*‐value of < 0.05 was considered statistically significant.

## 3 Results

Data analysis was performed encompassing 29 patients who met the inclusion criteria. Twenty‐one patients (72.4%) were male, and 8 patients (27.6%) were female. The majority of the patients were 56–60 years old, with the youngest patient belonging to the 26 to 30 age group and the oldest to the 81 to 85 age group. Clinical characteristics are shown in Table 1.

**TABLE 1:** Clinical characteristics.

		*n*	%
Sex	Male	21	72.4
	Female	8	27.6
Histology available		20	69.0
Psoriasis arthritis		5	17.2
*Comorbidity*			
	Chronic inflammatory bowel diseases	0	0
	Depression	2	6.9
	Chronic kidney disease	5	17.2
	Hepathopathy	1	3.4
	Cardiovascular diseases	12	41.4
	Malignancy	2	6.9
	Hepatitis B	2	6.9
	Hepatitis C	1	3.4
	Latent tuberculosis	2	6.9
	Alcohol abuse	4	13.8
	Nicotine abuse	6	20.7
*Academic center*	University Hospital Regensburg	4	13.8
	University Hospital Augsburg	2	6.9
	University Hospital Erlangen	10	34.5
	LMU Munich	7	24.1
	TU Munich	6	20.7

The average time between the first diagnosis of psoriasis and the occurrence of EP was 14.1 years (±15.9 years). In five patients (17.2%), erythroderma was the first clinical manifestation of psoriasis. Fourteen patients (48.3%) had been treated with phototherapy in the past for their PsO prior to the occurrence of EP. In nine cases (31%), PsO was treated with cDMARDs; four cases (13.8%) had been treated with biologics.

After the diagnosis of EP, systemic treatment was initiated after an average of 27.3 days (±40.0 days, median 12 days, minimum 0 days, maximum 191 days). In one patient, delayed re‐presentation to the treating center resulted in a prolonged interval between diagnosis and treatment initiation.

In eight patients (27.6%), cDMARDs were initiated after diagnosis of EP, and in 21 patients (72.4%), biologics were used. Biologic agents were administered according to approved dosing regimens (e.g., infliximab 5 mg/kg). Methotrexate doses ranged from 15 mg to a maximum of 20 mg subcutaneously once weekly. Fumarates were titrated according to standard escalation schedules up to a maximum dose of 720 mg. Cyclosporine was administered at approximately 2.5 mg/kg/day (maximum 100 mg daily). In some patients, temporary hospitalization with intensive topical therapy was used as supportive management during treatment initiation. Objective outcome measures, especially PASI values, were missing in eight patients because of non‐standardized follow‐up, incomplete records and continuation of care outside the participating centers. Further statistical analyses were performed in the subset of 21 patients with available objective outcome measures.

The mean PASI value at the start of systemic therapy was 31.9 (±17.1, range: 8–72). The average baseline DLQI value was 18.8 (±9.2, range: 3–30).

The mean time to the first PASI follow‐up was 26.1 weeks (±49.7 weeks, minimum 2 weeks, maximum 190 weeks).

The PASI value after initiation of systemic therapy was reduced to a mean of 10.8 points for all systemic therapies combined (Table 3). The PASI value was reduced by an average of 21.1 points (±19). There was a significant reduction in the Wilcoxon signed‐rank test (*p* < 0.001).

Patients receiving cDMARDs showed a mean PASI reduction of 25.3 points (*p* = 0.028) at first follow‐up. Patients receiving biologic therapy showed a mean PASI reduction of 19.4 points (*p* = 0.006) at first follow‐up (Figure 1). The DLQI improved on average to 5.7 (±7.9, minimum 0, maximum 20).

**FIGURE 1:**
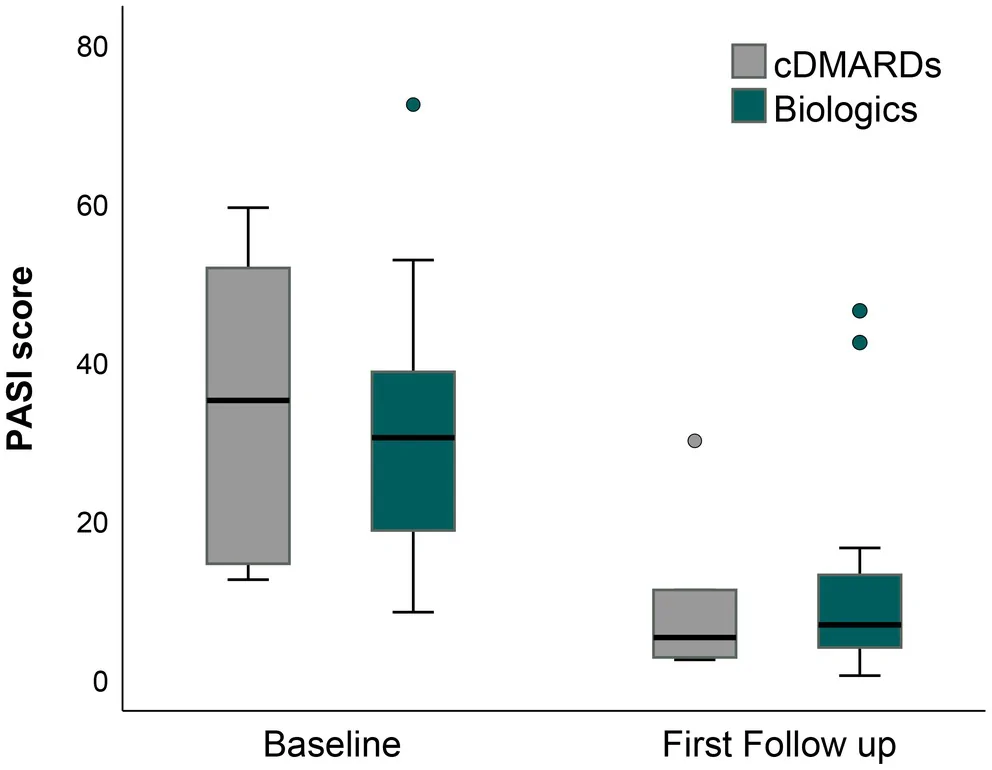
Boxplots of PASI scores at baseline and first follow‐up for cDMARDs and biologics.

PASI outcomes within the 8–16 week follow‐up window were available for 12 patients, including 10 treated with biologic agents (TNF‐alpha inhibitors [n = 2], IL12/23 inhibitors [n = 1], IL17 inhibitors [n = 4], and IL23 inhibitors [n = 3]) and two treated with conventional systemic therapy (methotrexate and fumarates). There was a mean PASI reduction of 19.9 points (*p* = 0.005).

Three patients with cDMARDs (one methotrexate, fumarates, and cyclosporine each) reached PASI 75 at any point during follow‐up. PASI 100 was not reached by cDMARDs. PASI 75 was achieved in 11 patients with biologic therapy (Table 2) at any point during follow‐up. Furthermore, four patients reached PASI 100. One patient was treated with infliximab, one with ustekinumab, and two with risankizumab (further results in Tables 2 and 3 and Figure 2). Side effects were noticed in seven patients (24.1%) (Table S1), including five of eight patients (62.5%) receiving cDMARDs and two of 21 patients (9.5%) receiving biologic therapy.

**TABLE 2:** Summary of EP patients with follow‐up parameters.

Pat.	Initiated treatment	Sex	Previous systemic treatment	Baseline PASI	PASI^a^	PASI^b^	PASI 75^c^	PASI 100^d^
1	*MTX*	M	MTX	59	29.6	29.6	no	no
2	*MTX*	M	N/A	51	6	N/A	***yes*** (w118)	no
3	*MTX*	F	—	33	N/A	N/A	N/A	N/A
4	*MTX*	M	N/A	N/A	N/A	N/A	N/A	N/A
5	*Fumarates*	M	Cs	14	11	N/A	no	no
6	*Fumarates*	M	—	12	4	4	no	no
7	*Fumarates*	M	—	42	2	N/A	***yes*** (w112)	no
8	*Cs*	F	—	28	2	N/A	***yes*** (w190)	no
9	*IFX*	M	ADA, CZP, BIM, IXE, SEC	30	4.2	0	***yes*** (w8)	***yes*** (w12)
10	*IFX*	M	Acitretin	20	46	46	no	no
11	*ADA*	M	—	15	N/A	N/A	N/A	N/A
12	*ADA*	F	—	26	N/A	N/A	N/A	N/A
13	*UST*	M	—	34	6	N/A	***yes*** (w4)	no
14	*UST*	M	—	13	5	0	***yes*** (w16)	***yes*** (w16)
15	*IXE*	M	—	72	7.5	7.5	***yes*** (w12)	no
16	*IXE*	M	—	35	N/A	N/A	N/A	N/A
17	*BIM*	M	MTX, GUS	40	N/A	N/A	N/A	N/A
18	*SEC*	F	MTX, Fumarates	42	42	42	no	no
19	*SEC*	F	Fumarates	23	2.9	0.6	***yes*** (w3)	no
20	*SEC*	M	—	41	12	2.5	***yes*** (w2)	no
21	*RIS*	M	N/A	17	0	0	***yes*** (w12)	***yes*** (w12)
22	*RIS*	F	—	36	5	N/A	***yes*** (w4)	no
23	*RIS*	M	Fumarates, ADA	15	0	N/A	***yes*** (w5)	***yes*** (w5)
24	*RIS*	F	APR	8	2	2	***yes*** (w12)	no
25	*TIL*	M	—	33	16	N/A	no	no
26	*TIL*	M	MTX, ADA	32	N/A	N/A	N/A	N/A
27	*TIL*	F	—	30	N/A	N/A	N/A	N/A
28	*GUS*	M	—	29	14	N/A	no	no
29	*GUS*	M	—	52	11.2	11.2	***yes*** (w11)	no

Abbreviation: PASI: Psoriasis Area and Severity Index.

^a^ PASI at first available follow‐up.

^b^ PASI follow‐up at 8–16 weeks.

^c^ ≥ 75% reduction in PASI from baseline at any time during follow‐up.

^d^ ≥ 100% reduction in PASI from baseline at any time during follow‐up; MTX: methotrexate; Cs: cyclosporine; IFX: infliximab; ADA: adalimumab; CZP: certolizumab; BIM: bimekizumab; IXE: ixekizumab; SEC: secukinumab; UST: ustekinumab; RIS: risankizumab; APR: apremilast; TIL: tildrakizumab; GUS: guselkumab; N/A: not available.

**TABLE 3:** Psoriasis area and severity index (PASI) response in different subgroups.

Therapy class	*n*	Baseline PASI	PASI at first follow‐up	Δ PASI at first follow‐up	*p*‐value
		Mean value (± SD) (Min–Max)	Mean value (± SD) (Min–Max)	Mean value (± SD)	Wilcoxon‐ test
**cDMARDs**	6	34.2 (± 17.8) (12–59)	9.1 (± 10.6) (2–30)	25.3 (± 16.8)	***p* = 0.028**
**Total biologics**	15	31 (± 16.7) (8–72)	11.6 (± 14.0) (0–46)	19.4 (± 20.1)	***p* = 0.006**
TNF‐alpha inhibitor	2	22.7 (± 6.4) (15–30)	25.1 (± 29.6) (4–46)	0.1 (± 36.6)	
IL12/23 inhibitor	2	23.4 (± 15) (13–34)	5.5 (± 1.3) (5–6)	17.9 (± 13.7)	
IL17 inhibitor	4	42.0 (± 16.3) (23–72)	16.1 (± 17.7) (3–42)	28.3 (± 26.9)	
IL23 inhibitor	7	28 (± 13.2) (8–52)	6.8 (± 6.7) (0–16)	20.3 (± 11.8)	
**Total**	21	31.9 (± 17.1) (8–72)	10.8 (± 12.9) (0–46)	21.1 (± 19)	***p* < 0.001**

**FIGURE 2:**
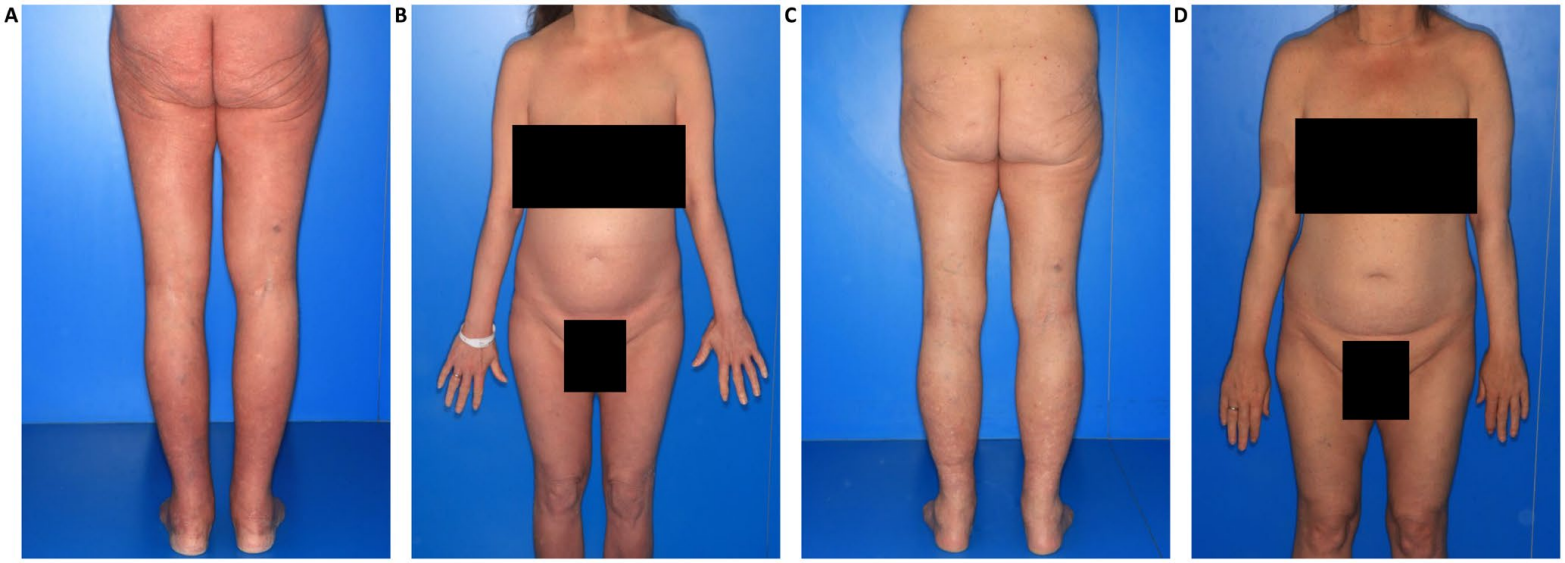
*A–B*, A Erythrodermic psoriasis in a 46‐year‐old female patient (October 2023). *C–D*, Clinical appearance 32 weeks after initiation of risankizumab 150 mg s.c., showing complete clearance (PASI 0).

In eight patients (27.6%) the selected systemic therapy was discontinued during the course of treatment. Methotrexate (2) and fumarates (2) were stopped because of side effects. In patients who were initially treated with infliximab (1), secukinumab (1), and guselkumab (1), there was a lack of efficacy. No reason was given for two patients (ixekizumab and risankizumab).

We performed a linear regression analysis to determine the relationship between different variables. We found a significant correlation between a history of phototherapy and baseline PASI. Patients with a history of phototherapy had a PASI score that was 16.28 points higher at the start of systemic therapy (*p* = 0.048). No abnormalities were found with regard to other clinical characteristics or previous medication.

Two patients died during the observation period, unrelated to systemic therapy and EP. One patient died 7 months and one patient 15 months after initiation of therapy.

## 4 Discussion

EP is a rare disease, typically affecting patients with a mean age of 48–55 years and showing a male predominance [3, 4]. In our cohort, we identified 29 patients across five study centers over a 5‐year period. Due to restrictions of the ethics approval concerning data protection, patient age was not recorded as exact values but categorized into age groups. The majority of patients were between 56 and 60 years of age, and 72.8% were male, supporting the notion that middle‐aged men are at increased risk of developing EP. We categorized EP as psoriasis with a BSA involvement of more than 75%. This threshold was chosen on the premise that, in a real‐world setting, the extent of affected body surface area cannot be assessed with the same accuracy as in prospective clinical studies. Applying this definition allowed us to establish a sufficiently large and consistent cohort for analysis.

EP arises from pre‐existing psoriasis in most patients. Prior studies reported rates of 78% and 96%, with a mean interval of 14 years [3, 21]. Our data are consistent, showing EP developing 14.1 years after initial PsO. Nonetheless, 17.2% of our patients presented with de novo EP, underscoring that this manifestation, although uncommon, does occur.

Regarding treatment effectiveness, cDMARDs (*n* = 8) reduced PASI by an average of 25.3 points (*p* = 0.028) in our cohort. PASI 75 was achieved in three patients (Table 2), whereas PASI 100 was not reached with any cDMARDs.

Cyclosporine is considered to be very effective in EP. In a cohort study of 33 EP patients receiving cyclosporine, complete remission was achieved in 67% of the patients. However, adverse events occurred in nearly 50% of patients, including eight drug‐related events [22]. In our cohort, only one patient received cyclosporine, possibly reflecting concerns about the potential severe side effects. Yet, PASI 75 was achieved in this case. Methotrexate was used in four patients; one achieved PASI 75, and two were lost to follow‐up. There is evidence supporting the use of methotrexate in EP. Collins et al. reported good or excellent responses to methotrexate in six of seven EP patients, without adverse events [23]. Kumar et al. described remission in two of three pediatric EP cases [24]. In our cohort, methotrexate was associated with the highest rate of adverse events among all treatment classes (75%), leading to treatment discontinuation in two cases. Therefore, although methotrexate may serve as an alternative treatment for EP, its potentially unfavorable safety profile should be carefully considered.

Among the three patients treated with fumarates, one patient reached PASI 75. Adverse events occurred in two patients (Table S1). To our knowledge, this is the first report describing fumarate effectiveness in EP.

Biologics lead to a mean PASI reduction of 19.4 points (*p* = 0.006) overall. PASI 75 was achieved in 11 patients, PASI 100 in four patients.

With regard to TNF‐alpha inhibitors, follow‐up data were available for two patients treated with infliximab. NPF guidelines recommend infliximab as a first‐line treatment option for EP [14]. Viguier et al. reported a ≥ 75% reduction in BSA in 48% of infliximab‐treated patients after 12–14 weeks [25]. In our cohort, one patient achieved PASI 100, whereas treatment was discontinued in the other patient because of insufficient efficacy. These observations suggest that infliximab may induce rapid and profound responses in some EP patients. However, efficacy appears variable, and non‐responders remain a relevant issue. Ustekinumab, an IL12/23 inhibitor, induced PASI 75 in both treated patients in our cohort, with one patient achieving PASI 100. In a larger case series of 22 EP patients, ustekinumab therapy led to PASI 90 in 68% of patients by Week 28, with sustained responses in most patients during long‐term follow‐up [26]. In contrast, Viguier et al. reported only limited efficacy, with one of three patients achieving PASI 50 [25]. Although our data are limited to two patients, they rather support the potential effectiveness of ustekinumab in EP.

In previous studies, IL17 blockade demonstrated high efficacy in EP with a good safety profile. In a cohort of eight ixekizumab‐treated patients, 100% achieved PASI 75 and five PASI 90 at Week 12 [27]. Similarly, in a study of 13 EP patients treated with secukinumab, a 77% response rate with 10 patients achieving PASI 90 was reported [28]. Avallone et al. compared both agents in 15 EP patients: although ixekizumab showed limited efficacy in this study, 82% of patients on secukinumab achieved PASI 90. There were no remarkable adverse events [29]. Follow‐up data were available in our study for four patients treated with IL17 inhibitors. One patient receiving ixekizumab and two of three patients receiving secukinumab achieved PASI 75, whereas none reached PASI 100. Our results are consistent with the current literature, albeit with smaller effect sizes, likely reflecting the limited sample size and real‐world treatment variability.

Among the biologics, IL23 inhibitors were most frequently used in our cohort (Table 2). Currently risankizumab data are limited but include a Japanese phase III randomized multicentre study of nine EP patients, all of whom achieved marked improvement and PASI 90 by Week 16 [30]. Similarly, guselkumab demonstrated high efficacy in a Japanese cohort of 11 patients, with a median improvement of 94% by Week 52 [31]. Evidence for tildrakizumab is restricted to two case reports, both describing complete clearance [32, 33]. In our cohort, IL23 inhibitors achieved a remarkable reduction of baseline PASI. Particularly risankizumab showed promising results, with all patients achieving PASI 75% and 50% achieving PASI 100 at any point during follow‐up. One of two patients treated with guselkumab achieved PASI 75, whereas no PASI 75 response was observed with tildrakizumab. However, follow‐up data were missing for two patients. The safety profile for IL23 inhibitors was favorable, with only one patient treated with risankizumab developing elevated liver enzymes (Table 3). These findings support IL23 blockade as a highly promising strategy in EP. Further prospective evaluation is necessary.

Altogether, 12 different agents were used as a first‐line therapy in our study. In eight cases, therapy was discontinued, and a second‐line therapy was initiated in five cases. This striking heterogeneity in treatment approaches, even among geographically close academic centers, highlights the absence of a uniform therapeutic strategy for EP. The variability not only reflects the lack of standardized treatment algorithms and evidence‐based guidelines but also emphasizes the clinical uncertainty physicians face when managing EP.

This study has several limitations, including its retrospective design with potential selection and reporting bias. Diagnosis of EP was primarily clinical and may have been uncertain given the challenging differentiation from other causes of erythroderma. In prospective studies, molecular assessments and transcription modules could be used to categorize inflammatory diseases and verify the diagnosis of EP [34]. The use of a > 75% BSA threshold may limit comparability with studies applying stricter erythroderma definitions. Treatment outcomes and follow‐up data were incomplete and heterogeneous, preventing consistent PASI assessments and may have led to under‐ or overestimation of treatment effects and adverse events. The overall small sample size, inherent to the rarity of EP, limits statistical power and generalizability of our findings. Finally, as the study population was derived from a single geographic region, the results may not be fully generalizable to other settings. Despite these limitations, our data provide valuable real‐world insights into the management of this rare and severe disease.

## Funding

The authors have nothing to report.

## Disclosure

Statistical Support: We thank Florian Zeman, PhD, biostatistician at the Center for Clinical Studies, University Hospital Regensburg, Germany, for statistical advice.

## Ethics Statement

Reviewed and approved by the ethics committee of the University of Regensburg (24–3742‐104), being valid for all Bavarian universities.

## Consent

The study was performed in accordance with the Helsinki Declaration of 1964 and its later amendments. Written informed consent for publication of clinical images was obtained from the patients depicted. For the retrospective analysis of anonymized clinical data, specific informed consent was not necessary.

## Conflicts of Interest

The authors have been an advisor and/or received speakers' honoraria or travel expense reimbursements and/or received grants and/or participated in clinical trials of the following companies/organizations: Julian Kögel: Almirall, Lilly, Novartis, Sanofi, UCB Pharma. Sandra Schuh: AbbVie, Almirall, Galderma, Eli Lilly. Julia Welzel: AbbVie, Almirall, BMS, Boehringer Ingelheim, Janssen, Leo, Lilly, Mibe, Novartis, Pfizer, Sana, and Sanofi. Michael Sticherling: AbbVie, Almirall, Amgen, BMS, Boehringer Ingelheim, Celgene, Johnson & Johnson, Leo, Lilly, MSD, Novartis, Pfizer, Regeneron, Sandoz, Sanofi, UCB. Carola Berking: Almirall, BMS, MSD, Pierre Fabre, Novartis, InflaRx, Immunocore, Delcath, SkylineDx, Regeneron, Sanofi. Felix Lauffer: AbbVie, Almirall, Amgen, Biogen, Boehringer Inglheim, Bristol‐Myers‐Squibb, Janssen, Johnson & Johnson LEO Pharma, Lilly, Novartis, Roche, Sanofi, Regeneron, UCB, Union Therapeutics, Hexal, Biogen. Tilo Biedermann: AbbVie, Alk‐Abelló, Almirall, Celgene‐BMS, Lilly, Mylan, Novartis, Phadia‐Thermo Fisher, Sanofi, Regeneron, Viatris. Susanne Bauer: Lilly. Michael Spindler: AbbVie. Mark Berneburg: AbbVie, Almirall, Amgen, Biofrontera, Biogen, Bristol‐Myers‐Squibb, Celltrion, Chiesi, CSL Behring, Galderma, Incyte, Janssen‐Cilag, LEO Pharma, L'Oréal, Medicos Kosmetik, MSD, Mylan, Novartis, Pfizer, Pierre‐Fabre, Sanofi, Stadapharm, Takeda Pharma, UCB Pharma. Dennis Niebel: AbbVie, Almirall, AstraZeneca, Biogen, Boehringer Ingelheim, Bristol‐Myers‐Squibb, Celltrion, GlaxoSmithKline, Incyte, Janssen‐Cilag, Kyowa Kirin, LEO Pharma, Lilly, L'Oréal/Cerave, MSD, Novartis, Pfizer, Regeneron, SUN Pharma, and UCB Pharma. No potential conflicts of interest were disclosed by the remaining authors.

## Supporting information


**Table S1:** Side effects.

## Data Availability

The datasets generated during the current study are available from the corresponding author on reasonable request.
