# Supplementary material for: Systemic Treatment and Outcome in Erythrodermic Psoriasis: A Retrospective Multicenter Study
Source: Int J Dermatol. 2026 Apr 17;65(12):2524–31. doi: 10.1111/ijd.70422 (PMC13745617; doi:10.1111/ijd.70422)
Supplement: Supplementary file 1 [file IJD-65-2524-s001.docx]

Supplementary Tables

| *Initiated treatment EP* | *Side effects* | *Discontinuation* |
| --- | --- | --- |
| *MTX* | Elevated liver enzymes | yes |
| *MTX* | Elevated liver enzymes | no |
| *MTX* | Nausea, vomiting | yes |
| *Fumarates* | Flush | no |
| *Fumarates* | Flush, lymphocytopenia | yes |
| *IXE* | Upper airway infections, Nausea, Headache | no |
| *RIS* | Elevated liver enzymes | no |
| MTX: methotrexate; IXE: ixekizumab; RIS: risankizumab | | |

**Tab S1:** Side effects
